# Genetic Diversity and Prevalence of Porcine Circovirus Type 2 in China During 2000-2019

**DOI:** 10.3389/fvets.2021.788172

**Published:** 2021-12-15

**Authors:** Ning Li, Jing Liu, Jiali Qi, Feng Hao, Lei Xu, Kangkang Guo

**Affiliations:** ^1^College of Veterinary Medicine, Northwest A&F University, Yangling, China; ^2^College of Life Sciences, Northwest A&F University, Yangling, China

**Keywords:** PCV2, nucleotide sequence, genetic analysis, cap protein, vaccine

## Abstract

As the major pathogen for porcine circovirus-associated disease (PCVAD), porcine circovirus type 2 (PCV2) is no longer treated as an emerging virus anymore. The wide distribution of PCV2 infection in China causes huge economic losses in the swine industry. Currently, it is generally believed that PCV2 has eight genotypes (PCV2a to PCV2h), with PCV2a, PCV2b, and PCV2d being widely distributed. To comprehensively explore the genetic diversity and prevalence of PCV2 in China, PCV-2 sequences submitted from China in the GenBank database were retrieved. With a total of 714 PCV2 strains were retrieved, we found that early-submitted PCV2 sequences were mainly collected from coastal provinces in the southeast part of China, which may indicate PCV2 was initially circulating in those regions. From 2002 to 2008, PCV2b was the dominant prevalent genotype in those retrieved sequences. From 2009, PCV2d became the dominant genotype in those sequences, dropping a hint that a potential shift of PCV2b to PCV2d might occur in 2009, which is similar to the patterns at the global level. In addition to the PCV2a, PCV2b, and PCV2d genotypes, novel strains were also characterized. We further revealed that the amino acid sequences consistency of PCV2a Cap is higher than those in other genotypes. Together, this study provided clues for the possible prevalent genotypes and dynamics of genetic diversity in China from 2000 to 2019.

## Introduction

As the smallest non-enveloped virus, porcine circoviruses (PCVs) have a circular, single-stranded DNA genome. They are members of the genus *Circovirus* in the *Circoviridae* family (1). First Porcine circovirus-associated disease (PCVAD) was reported in 1998 and the pathogen was characterized as porcine circovirus type 2 (PCV2) (2–4). In addition to PCV2, other species of PCVs including porcine circovirus type 1 (PCV1) and porcine circovirus type 3 (PCV3) were also identified (1, 5). Recently, a novel circovirus PCV4 was first reported in Hunan province, China (6) and later in other provinces of China (7, 8).

Currently, eight genotypes (PCV2a-PCV2h) have been identified (9). A nucleotide diversity cut-off of 3.5% was used to define different PCV2 genotypes. PCV2a and PCV2b were identified in 2008 (10) and retrospectively study characterized PCV2c from Denmark (11). Recent studies indicated this genotype also circulating in feral pigs from Brazil and in China (12, 13). Subsequently, a fourth genotype named PCV2d has emerged (14) and this genotype is prevalent worldwide. Increasingly new genotypes have been identified like PCV2e was identified from the USA and Mexico (15, 16). Furthermore, PCV2f, which has a novel viral sequence clustering that does not match the existing genotypes was identified in China (17). The distribution of PCV2g was reported in Austrian pigs in a sample from 2009 and also in the India pig population (18, 19). PCV2h was detected in China and Korean (20, 21).

It is commonly believed the length of the PCV2 genome is about 1,767 nucleotides (nt), with six open reading frames (ORFs) have been characterized (22). The ORF1 and ORF2 encode replicase (Rep) and capsid (Cap) proteins are major ORFs (23). ORF2 encoded Cap protein is the major structural protein that responsible for viral entry and triggering neutralization antibodies production. Compared with other ORFs, the ORF2 gene can be used as a phylogenetic and epidemiological marker due to its high genetic variations. For instance, compared with PCV2a-2d, the ORF2 sequence of PCV2e has 12 or 15 additional nts (CTT/TCTTATATGTAA) (24).

In China, the first identification of PCV2 in pig farms was reported in 2000 and increasing prevalence was reported after that (14, 25–29). Although remarkable knowledge of the epidemiology of PCV2 infection in China has been obtained (20, 30), there are few studies focusing on the genetic diversity and prevalence of PCV2 from the emerging of this important virus. In this study, we collected and analyzed the sequences from GenBank that were submitted from China between 2001 and 2019. Based on those sequences, we suggested that PCV2 genotypes in China are undergoing constant changes and the predominant strains were shifted to PCV2d in recent years.

## Materials and Methods

### Sequence Collection

Seven hundred and fourteen complete PCV2 genomes, collected between 2001 and 2019 from different provinces in China, were retrieved from GenBank at the National Center for Biotechnology Information (https://www.ncbi.nlm.nih.gov/). ORF2 of these sequences was analyzed by ORFfinder in NCBI.

### GenBank Accession Numbers

The GenBank accession numbers of 714 PCV2 genomes retrieved from NCBI are listed in Supplementary Table 1. The reference sequences of PCV2a (Accession: KY810321.1 and KY940532.1), PCV2b (Accession: KX831482.1 and MF150189.1), PCV2d (Accession: MF169693.1 and MF169747.1) and genotypes were obtained.

### Sequence Alignment and Phylogenetic Analysis

All sequences were aligned by ClustalW using MEGAX. The maximum likelihood (ML) fits of 24 different nucleotide substitution models were used to find the most suitable parameters of the phylogenetic tree. Models with the lowest BIC scores (Bayesian Information Criterion) are considered to describe the substitution pattern the best. Phylogenetic trees were constructed using ORF2 sequences of each complete sequence, by using the maximum-likelihood method in MEGAX. The alignment sequence was used to construct the phylogenetic tree with MEGAX, and the parameters were as follows: the statistical method is ML, the test of phylogenetic is the bootstrap method, the number of bootstrap replications is 1,000, the gaps or missing data treatment is partial deletion and the ML heuristic method is Nearest-Neighbor-Interchange (NNI).

### Amino Acid Sequence Alignment of Cap Protein

15 sequences of PCV2a, PCV2b and PCV2d were selected randomly for each genotype and two PCV2e sequences found in 2017 were also included and amino acid sequences of Cap protein were generated by using Lasergene software (DNASTAR, Madison, WI, USA). Next, the amino acid sequence difference between those sequences was compared Based on the Molecular Evolutionary Genetics Analysis X (MEGA X).

### Possible Spatial Structure Analysis

To analyze the possible spatial structure analysis of Cap protein of different genotypes, the consensus amino acid sequences of different genotypes were obtained by Megalign in Lasergene software (DNASTAR, Madison, WI, USA). The protein sequences of different genotypes to DNAMAN 8.0 (Lynnon Biosoft, Foster City, CA, USA) and then the possibility of different secondary structures at different locations were analyzed.

## Results

### The Prevalence of PCV2 in China During the Period 2000-2019

**Figure 1:**
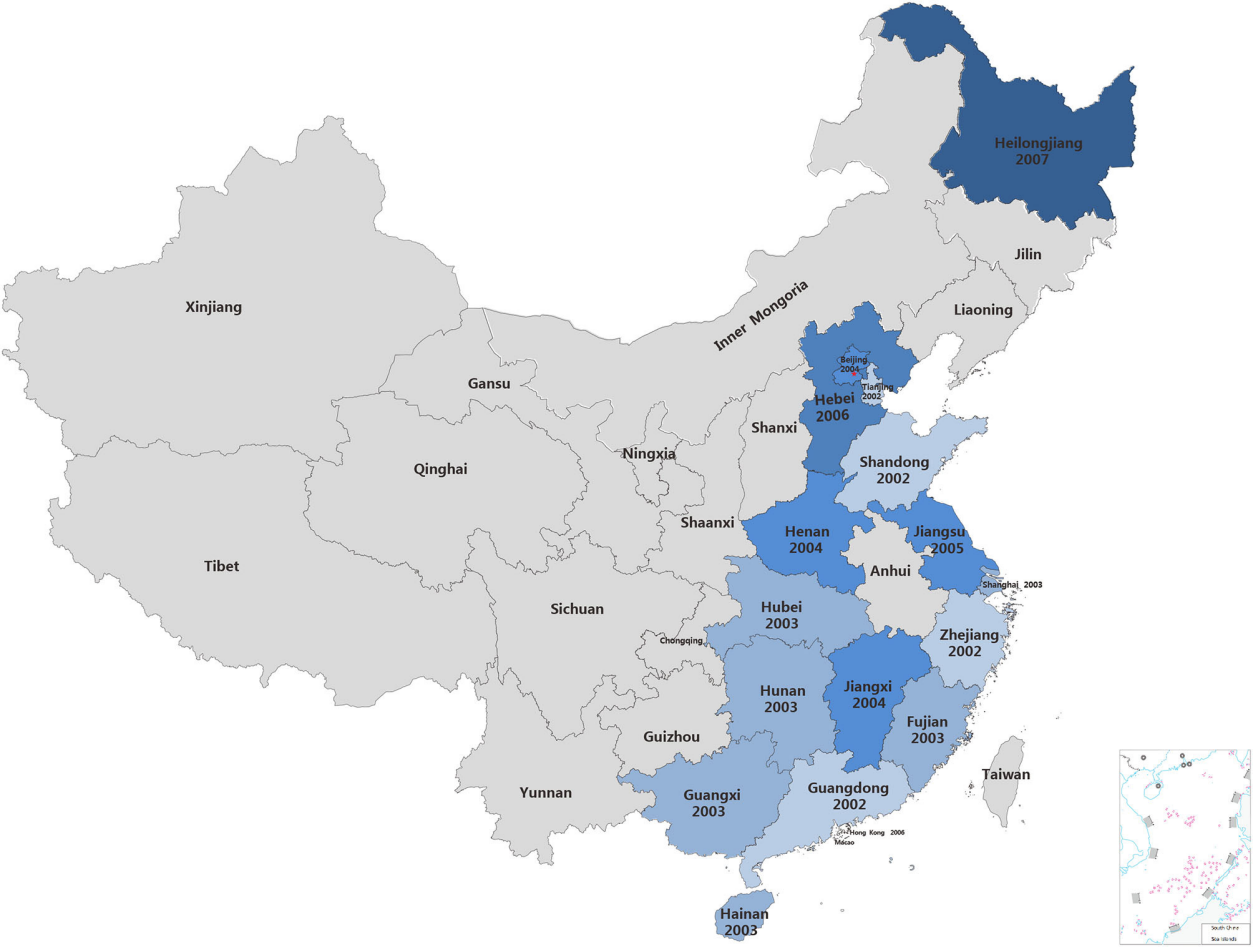
The detection PCV2 in China. The year on the map indicate the time when PCV2 sequences were submitted for the first time in these provinces.

To explore the transmission of PCV2 in China, PCV2 sequences deposited in the GenBank database were retrieved. A total of 714 PCV2 strains were collected from the GenBank that were submitted from China between 2001 and 2019 (Supplementary Table 1). In 2001, four sequences of PCV2 strains were collected from the GenBank. However, the location that collected these strains was not mentioned. In 2002, sequences for PCV2 strain collected from four coastal provinces including Guangdong, Shandong, Tianjin and Zhejiang provinces were submitted to GenBank. In 2003, PCV2 infection was mentioned in Hubei, Shanghai, Fujian, Guangxi, Hainan and Hunan provinces, which are coastal provinces or next to the provinces that already have PCV2 outbreaks. Next year, PCV2 infection spread to Jiangxi, Beijing and Henan provinces. After that, PCV2 gene sequences in GenBank suggested PCV2 infection might spread in Jiangsu, Hebei provinces (2005), Hong Kong (2006) and the northeast part of China, Heilongjiang (2007) (Figure 1). Thenceforth, an enormous amount of PCV2 sequences were submitted from China (Supplementary Table 1). Based on this chronological information, we speculated that PCV2 may initially circulate in coastal provinces in the southeast part of China and then spread to other provinces.

### Phylogenetic Analysis of PCV2 Strains

**Figure 2:**
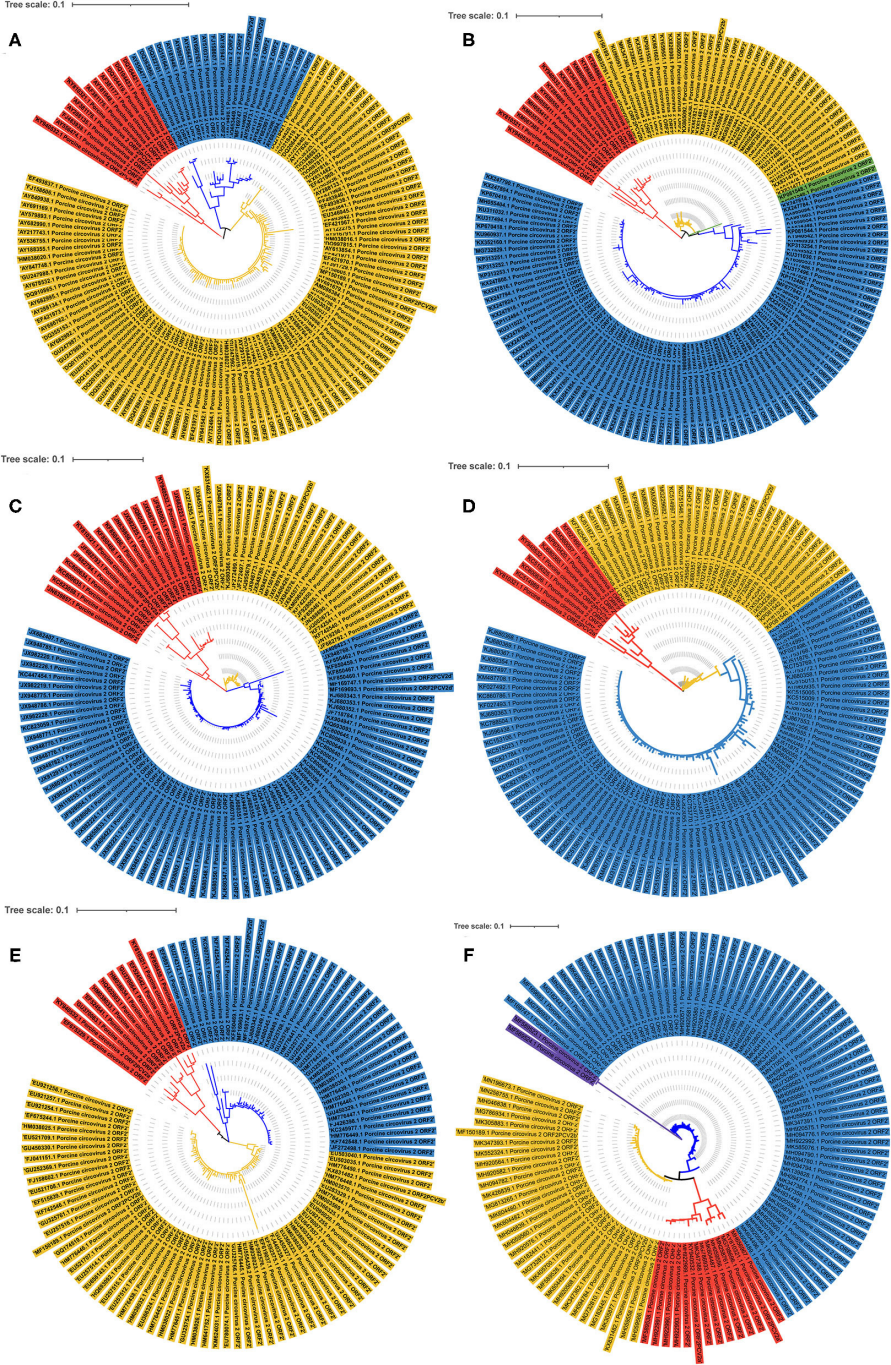
The phylogenetic ML tree of ORF2 of PCV2 strains. The phylogenetic ML tree of ORF2 from PCV2 strains deposited in GenBank from China between 2001 and 2019. **(A)** ML tree of ORF2 from PCV2 strains from 2001 to 2006. **(B)** ML tree of ORF2 from PCV2 strains from 2007 to 2009. **(C)** ML tree of ORF2 from PCV2 strains from 2010 to 2011. **(D)** ML tree of ORF2 from PCV2 strains from 2012 to 2013. **(E)** ML tree of ORF2 from PCV2 strains from 2014 to 2016. **(F)** ML tree of ORF2 from PCV2 strains from 2017 to 2019. Different colors indicate different genotypes. Red, PCV2a; Yellow, PCV2b; Blue, PCV2d; Purple, PCV2e; Green, PCV2b+2d. The tree was constructed by the maximum likelihood method with bootstrap values calculated for 1,000 replicates.

To more comprehensively understand the extent of genetic diversity of PCV2 in China, 714 PCV2 strains sequences in GenBank were analyzed by phylogenetic analysis. Phylogenetic construction was conducted using ORF2 sequences. As shown in Figures 2A,B, from 2001 to 2006, the PCV2a was the most prevalent genotype while PCV2d may become the second prevalent genotype from 2007 to 2009 in those retrieved sequences. Of note, after 2009, PCV2d became the dominant strain while PCV2a is the minority in those sequences (Figures 2C,D). From 2014 to 2016, two strains that were recombined by PCV2b and PCV2d were identified (Figure 2E) and from 2017 to 2019, novel PCV2e was identified in those sequences (Figure 2F).

### The Prevalence of PCV2 Genotypes in China

**Figure 3:**
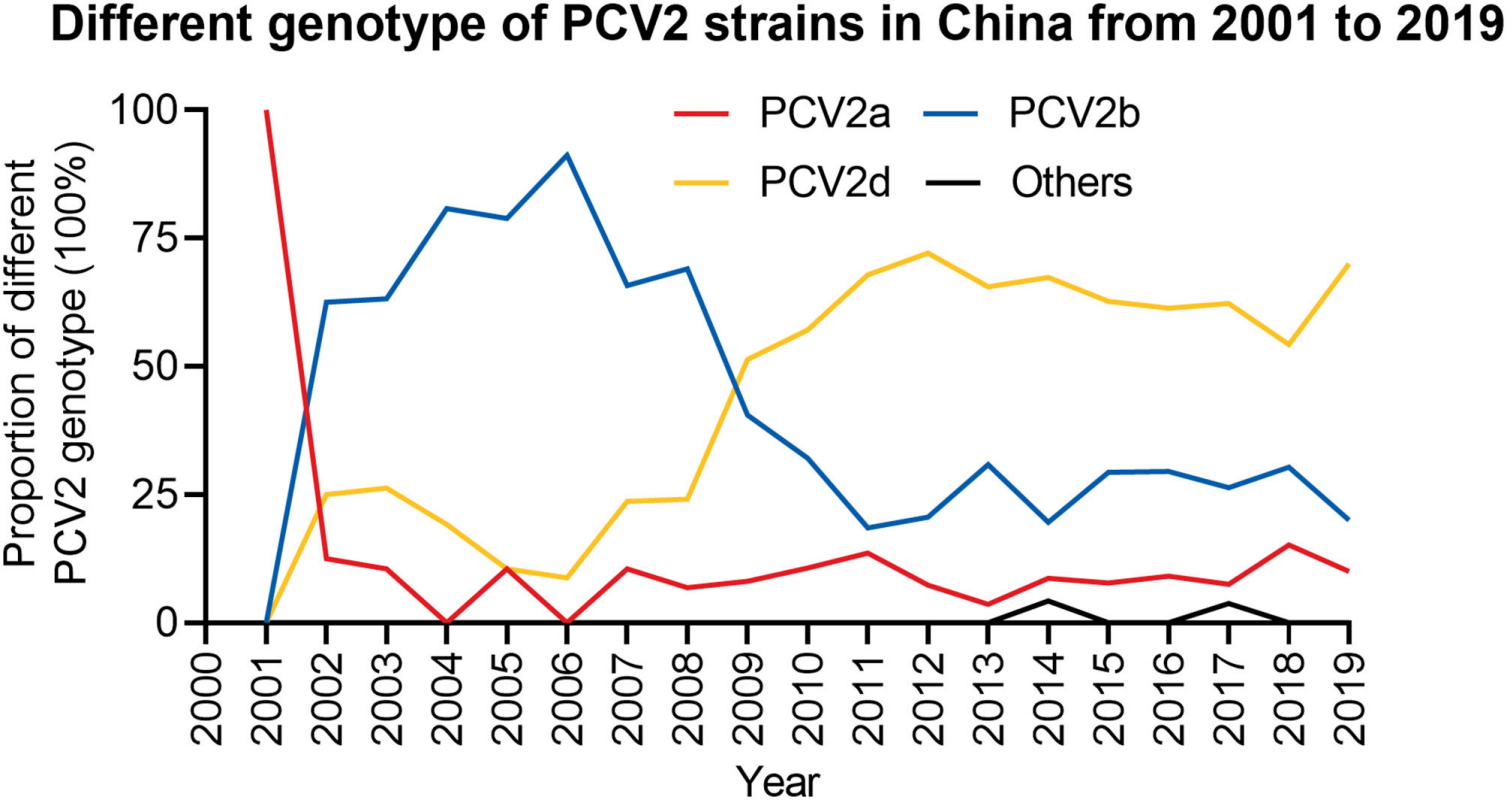
The proportion of different PCV2 genotypes from 2001 to 2019. The proportion of different PCV2 genotypes in each year was calculated. Red, PCV2a; Blue, PCV2b; Yellow, PCV2d; Black, Others.

To further explore the prevalence of PCV2 in China, the genotype of 714 PCV2 strains collected that from GenBank were analyzed. Between 2001 and 2019, the main prevalent PCV2 genotypes in those sequences were PCV2a, PCV2b and PCV2d (Figure 3 and Supplementary Table 2). By analyzing the complete genome sequences, we showed that all sequences summited in 2001 were belong to genotype PCV2a. However, this did not suggest no PCV2b and PCV2d was circulating at the time since only four sequences were collected. From 2002 to 2008, PCV2b was the dominant prevalent genotype in those sequences, while the prevalence of PCV2a was lower than PCV2d. During this time, the proportion of PCV2d witnessed a fluctuation but its overall tendency was increased. From 2009, PCV2d become the predominant genotype in those sequences, while the proportion of the PCV2b in all sequences was substantially decreased. Of note, PCV2a was still the lowest prevalent strain in those sequences during this period. The analysis of those sequences may suggest PCV2d is still the main epidemic strain currently and the prevalence of PCV2a, PCV2b, and PCV2d are rather stable from 2009. Specifically, the proportion of PCV2a in all retrieved sequences at that year was around 10%, while the proportion of PCV2b was around 20-30% and PCV2d was <70%.

Although these three genotypes (PCV2a, PCV2b, and PCV2d) were the most widely detected strains in those retrieved sequences from the report of PCV2 infection, novel strains were also characterized. In 2014 and 2017, some sequences that did not belong to those major genotypes were also identified. The new genotype identified in 2014 was the recombinant of PCV2b and PCV2d, and in 2017, the emerged genotype was PCV2e (Figure 3 and Supplementary Table 2), which was first reported in the United States in 2015 with a 702 bp ORF2 (15, 31).

### Amino Acid Sequence Differences of PCV2 Cap Protein

**Figure 4:**
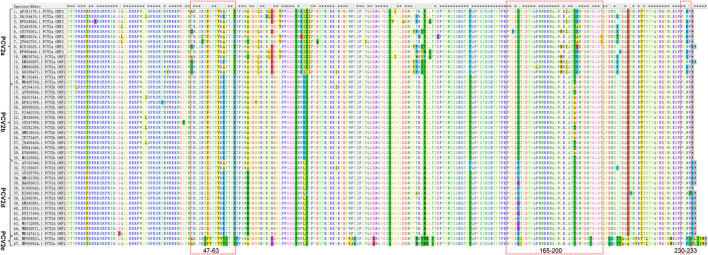
Amino acid sequence differences of PCV2 Cap protein. 15 PCV2a, 15 PCV2b, 15 PCV2d, and 2 PCV2e were selected respectively, and their amino acid sequences were compared by using the Alignment. The Alignment was performed using the software MegAlign, the major amino acid mutations are displayed in a box. The overlapping antigen sites (47-63, 165-200, and 230-233) of Cap protein among different genotypes are indicated by red dashed boxes.

To further characterize differences of Cap protein among PCV2a, PCV2b, PCV2d, and PCV2e, amino acid sequence alignment was performed. We showed that the length of Cap protein varies in different genotypes (Figure 4). Most of the PCV2a and PCV2b strains have a Cap protein with 233 amino acids, while the majority of PCV2d strains have a Cap protein with 234 amino acids. Of note, many PCV2e strains have a larger Cap protein with 238 amino acids. Amino acid sequences similarity of Cap within specific genotype are high. Particularly, amino acid sequences consistency of PCV2a Cap is greater than that in other genotypes. However, significant differences in Cap protein amino acid sequence were observed between PCV2e and other genotypes.

**Figure 5:**
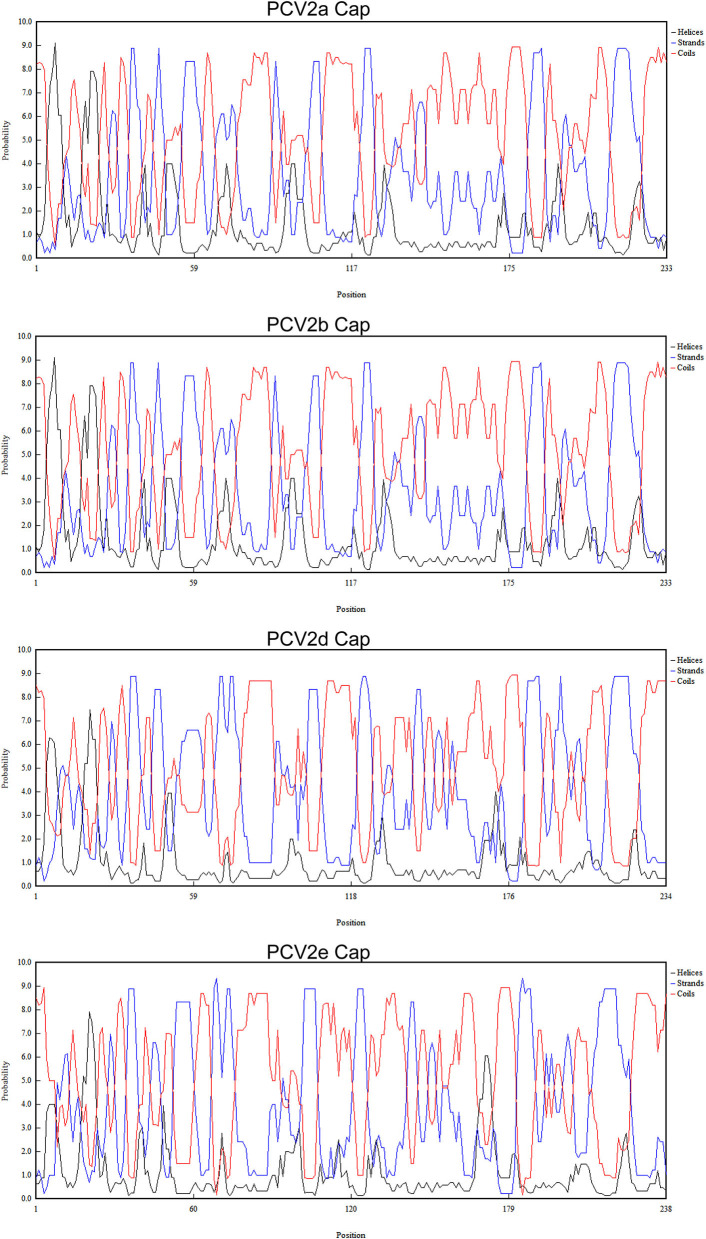
Predicated spatial structure of PCV2 ORF2 of different genotypes. The possible spatial structure of PCV2 ORF2 of different genotypes as helices, strands and coils.

To explore whether those essential residues are changed in different PCV2 genotypes, we compared the amino acid residues of PCV2a, PCV2b, and PCV2d. As shown in Table 1, amino acid sequences of three spatially overlapping antigen sites 47-63, 165-200, and 230-233 of Cap protein are largely different among different genotypes. Finally, analysis of the possible spatial structures of different genotypes revealed PCV2a and PCV2b showed high similarity while significantly different were observed PCV2d and PCV2e (Figure 5).

**Table 1:** Amino acid sequences difference among PCV2a, PCV2b, and PCV2d.

**AA position**	**PCV2a**	**PCV2b**	**PCV2d**
8	Y/F	Y	F
53	F	F	I
57	V	I	V
59	A	R/K	K
63	R/T	K/R	R
68	A	A	N
76	L/I	I	I
77	D/N	N	N
80	V/L	L	L
86	T	S	S
88	K	P	P
89	I	R	T
90	S	S	I
91	I	V	V
121	S/T	S	T
130	V/F	V	V
131	T/P	T	T
133	A/S/V	A	A
134	T	T	N
151	P	T	T
169	S	S	R/G
185	L/M	L	L
187	L/I	L	L
190	S	A	T
191	R/K/A	G	G
206	K/I	I	I
210	D	E	D
215	V	V	I
232	K	N	N
234	^*^	^*^	K

* *means termination codon*.

## Discussion

Porcine circovirus 2 (PCV2) is no longer treated as an emerging virus anymore, due to its widespread worldwide. The first report in 1998 (2–4), tremendous attention has been drawn by this virus, since its infection causes huge economic losses in the swine industry, including China. The first report of the postweaning multisystemic wasting syndrome (PMWS) related to PCV2 infection in China was published in 2000 (25). Then, PCV2 was detected in many tissue samples of the diseased pig (32). In this study, we retrieved PCV2 sequences from GenBank that were submitted from China between 2001 and 2019. Atm the early period, PCV2 sequences were mainly collected from Guangdong, Shandong, Tianjin, and Zhejiang provinces, all of which are coastal provinces in China. The submission of PCV2 sequences from inner provinces in China happened at later times, which may suggest the PCV2 was spread from the coastal provinces, perhaps following major swine commercial routes.

By analyzing those retrieved sequences, we noticed that between 2002 and 2008, the dominant prevalent genotype is PCV2b, which is consistent with the previous report (29). Afterward, although the PCV2 vaccines were widely used, the prevalent genotype may shift from PCV2b to PCV2d. Such genotype shift seems occurred globally and the worldwide use of PCV-2 vaccines may be the driven force (33, 34). Until now, this genotype is widely circulated and has become the predominant genotype worldwide (35–39). In our previous study, we showed that 38 PCV2 positive samples were detected from 300 blood samples collected from the slaughterhouse in Shaanxi province from 2018 to 2019. Of note, genotyping of those PCV2-positive samples showed that 11 samples were PCV2b positive out of 20 sequenced samples, while nine samples were PCV2d positive (40). However, this may only partially reflect the prevalence of PCV2 in Shaanxi due to a small number of samples being obtained. Recently, a study collected clinical samples from Henan province of China and the whole genome sequences of 20 PCV2 strains were analyzed (41). The results showed that among those 20 strains, 30% (6 strains) belonged to PCV2a, 30% (6 strains) belonged to PCV2b and 40% (8 strains) belonged to PCV2d. In another study, they complete genome sequences of 34 PCV2 strains that were collected from 55 different pig farms in central China from 2018 to 2020 (42). Consistently, among those 34 strains, 10 strains belonged to PCV2b and 16 strains belonged to PCV2d and only 8 strains belonged to PCV2a. Taken together, those observations implied that current dominant prevalent genotypes in China are PCV2b and PCV2d. Furthermore, genotype PCV2e was detected in 2017 in the Fujian province in China (31). Although the prevalence of this genotype was rare in our analysis (Figure 2) and was not detected in 300 blood samples collected in Shaanxi province, attention should be paid to this genotype. Importantly, the genotypes shift we showed in this study may not precisely reflect the reality that happened in the field and further clinical samples should be sequenced to determine and predict the circulation of different PCV2 genotypes.

As the principal strategies to control PCV2 infection, vaccination is broadly employed in the field. A commercial vaccine against PCV2a was first developed in the USA with efficient effects in alleviating the viral loads and clinical signs (43, 44). Despite different PCV2 genotypes has been circulating, most commercial vaccines are still generated based on PCV2a (44, 45). In particular, although one study has been reported that the PCV2a-based vaccine is capable to reduce PCV2d infection (46), PCV2d genotype was emerging reported worldwide (47–51). Compared with PCV2a and 2b, PCV2d are more virulent with severe clinical signs, viremia and lesions (52, 53). In line with these studies, we also revealed a potential shift of PCV2b to PCV2d from 2009 (Figure 3), which is similar to the patterns at the global level. Therefore, efforts should be taken to develop new commercially PCV2 vaccines based on the PCV2d genotype, but not PCV2a, since this genotype is probably no longer the predominant circulating genotype in China. The current PCV2a based vaccines should be evaluated for their ability to prevent the infection or transmission of other genotypes.

It has been reported that position 1-41 of Cap protein, a special arginine-rich N-terminus, the nuclear localization signal (NLS) that responds for its nucleus accumulation (54, 55). In particular, positions 12-18 and 34-41 of the amino acid residues are important for the nuclear localization of Cap protein (54). Amino acids 143-145 (N143YS) is the N-linked glycosylation site PCV2 Cap protein (56). The mutation of those sites leads to a higher level of immune responses and improved immunogenicity of Cap-based vaccination and stronger T lymphocyte proliferative activity and cytokine production (56), indicting those conserved sites might responsible for the failure of PCV2 vaccination. In addition, amino acid residues 47-63, 165-200, and 230-233 are characterized as essential for epitope recognition of PCV2 (57). Intriguingly, positions 169, 185, 187, 190, 191, and 232 in PCV2a were different from PCV2b and 2d and such substitutions may relate to the increased circulating of PCV2b and PCV2d in the PCV2a-vaccinated fields. Particularly, it has been proved that the Alanine (A) in Cap protein at position 59 of PCV2a genotype is an essential conformational neutralizing antibody epitope (58). Notably, this site was changed to R/K in PCV2b and K in PCV2d (Table 1), which might lead to a reduced recognition of this epitope in these two emerging genotypes. Consequently, animals that were vaccinated by PCV2a-based vaccine may have decreased antibody neutralizing ability against PCV2b infection.

PCV2a and PCV2b strains encode a Cap protein with 233 amino acids and PCV2d strains have a Cap protein with 233 or 234 amino acids. Of note, many PCV2e strains have extra12 nucleotides at the carboxyl terminus of ORF, which encodes Cap protein with 238 amino acids. A possible explanation of the extra length of the PCV2e capsid is that it is the prototype and a 12 nt deletion generated the progenitor of PCV2a to PCV2d (16). Importantly, such extra carboxyl terminus seems not to affect the predicted protein structure of PCV2e capsid protein (16). The different length between the PCV2a/PCV2b Cap protein and some of the PCV2d Cap protein is due to one extra Lysine (K) at the carboxyl terminus (Figure 4).

Cap protein is the main determinant of the PCV2 antigenicity and immunogenicity and the mutation of Cap protein may facilitate PCV2 to evade the host immune response. A single amino acid mutation in Cap protein led to a strain that can not be neutralized by currently available antibodies (58). The PCV2 nucleotide substitution was estimated to be 1.2 × 10^−3^ sites per year, which is extremely high for a single-stranded DNA virus (59). With such high evolution rates, prevalent genotypes are constantly varying and difficulties for vaccine development were substantially increased. In this study, we found that amino acid residues 47-63, 165-200, and 230-233, which are essential for epitope recognition of PCV2, are largely different among different genotypes (57). Further investigations regarding the antigens and protein structure variations of different PCV2 genotypes could pave the road for developing the new vaccine and deepen the understanding of PCV2 pathogenesis. In addition, co-infections of two different PCV2 genotypes in the field were also reported (60). In China, co-infection of different PCV2 genotypes (61) and even co-infection of PCV2 and PCV3 were also reported (62). Therefore, a better understanding of the molecular epidemiology of PCV2 is vital for controlling further infections.

In conclusion, by analyzing PCV2 strains collected from GenBank between 2001 and 2019 in China, we may speculate that PCV was initially circulating in coastal regions and spread to other provinces soon afterward. Furthermore, genotyping analysis of these sequences provides clues that PCV2b might be the dominant prevalent genotype from 2002 to 2008 and a potential shift to PCV2d might occur in 2009. Finally, we found amino acid sequences consistency of PCV2a Cap is greater than those in other genotypes. These results highlighted prevalent genotypes and dynamics of genetic diversity in China from 2000 to 2019, which is essential for the understanding of molecular epidemiology of PCV2-related diseases and the development of new PCV2 vaccines.

## Data Availability Statement

The original contributions presented in the study are included in the article/Supplementary Material, further inquiries can be directed to the corresponding author/s.

## Author Contributions

KG had the idea for the article. NL and LX analyzed the data. JL, JQ, and FH helped to analyze the data. KG and LX wrote the draft of the manuscript and critically revised the work. All authors contributed to the article and approved the submitted version.

## Funding

This work was supported by grants from the National Natural Science Foundation of China (Project No. 31672580).

## Author Disclaimer

Frontiers Media SA remains neutral with regard to jurisdictional claims in published maps and institutional affiliations.

## Conflict of Interest

The authors declare that the research was conducted in the absence of any commercial or financial relationships that could be construed as a potential conflict of interest.

## Publisher's Note

All claims expressed in this article are solely those of the authors and do not necessarily represent those of their affiliated organizations, or those of the publisher, the editors and the reviewers. Any product that may be evaluated in this article, or claim that may be made by its manufacturer, is not guaranteed or endorsed by the publisher.
