# Supplementary material for: Genetic Diversity and Prevalence of Porcine Circovirus Type 2 in China During 2000-2019
Source: Front Vet Sci. 2021 Dec 15;8:788172. doi: 10.3389/fvets.2021.788172 (PMC8717868; doi:10.3389/fvets.2021.788172)
Supplement: Supplementary file 1 [file Data_Sheet_1.docx]

**Supplementary Tables**

**Table S1. Accession number of PCV2 strains used in this study.**

| Year | Accession No. | | | | | | Seq. No. |
| --- | --- | --- | --- | --- | --- | --- | --- |
| 2001 | AF381175 | AF381176 | AF381177 |  |  |  | 3 |
| 2002 | AF538325 | AY122275 | AY177626 | AY181945 | AY181946 | AY181947 | 8 |
|  | AY181948 | AY188355 |  |  |  |  |  |
| 2003 | AY217743 | AY288133 | AY288134 | AY288135 | AY291316 | AY1291317 | 19 |
|  | AY291318 | AY294310 | AY391729 | AY510375 | AY536755 | AY536756 |  |
|  | AY556473 | AY556474 | AY556475 | AY556476 | AY556477 | AY578327 |  |
|  | AY579893 |  |  |  |  |  |  |
| 2004 | AY596822 | AY596823 | AY604430 | AY613854 | AY641542 | AY651850 | 26 |
|  | AY678532 | AY682990 | AY682991 | AY682992 | AY682993 | AY682994 |  |
|  | AY682995 | AY682996 | AY682997 | AY686762 | AY686763 | AY686764 |  |
|  | AY686765 | AY691169 | AY691679 | AY732494 | AY847748 | AY849938 |  |
|  | EU346945 | HM038020 |  |  |  |  |  |
| 2005 | AM086384 | AY916791 | AY943819 | AY969004 | DQ017036 | DQ104419 | 38 |
|  | DQ104420 | DQ104421 | DQ104422 | DQ104423 | DQ141322 | DQ151643 |  |
|  | DQ180392 | DQ180393 | DQ195679 | DQ201639 | DQ201640 | DQ201641 |  |
|  | DQ201642 | DQ206444 | DQ218419 | DQ218420 | DQ218421 | DQ322701 |  |
|  | DQ346683 | DQ355153 | DQ363860 | EU503031 | EU503036 | EU503037 |  |
|  | FJ483938 | GU247987 | GU247989 | GU247990 | GU247992 | HM038016 |  |
|  | HM038018 | HM038019 |  |  |  |  |  |
| 2006 | DQ478947 | DQ910865 | DQ910866 | DQ997815 | DQ997816 | DQ997817 | 34 |
|  | EF210106 | EF421967 | EF421968 | EF421969 | EF421970 | EF421971 |  |
|  | EF421972 | EF421973 | EF493837 | EF493838 | EF493839 | EF493840 |  |
|  | EF493841 | EF493842 | EU257513 | EU503038 | EU503039 | FJ158603 |  |
|  | FJ158605 | FJ158606 | FJ158607 | GU247988 | GU247991 | HM038021 |  |
|  | HM038027 | JQ653449 | JX679498 | KM604667 |  |  |  |
| 2007 | EF515839 | EF524541 | EF524542 | EF592575 | EF592576 | EF619037 | 38 |
|  | EF675229 | EF675244 | EF989713 | EU095020 | EU257512 | EU257514 |  |
|  | EU257515 | EU257516 | EU274311 | EU247312 | EU366323 | EU366324 |  |
|  | EU366325 | EU503035 | EU503040 | EU521707 | EU521708 | EU647557 |  |
|  | FJ041151 | FJ158602 | FJ426398 | GU252370 | HM038023 | HM038036 |  |
|  | HM038029 | HM038031 | HM038033 | HM641752 | KF742542 | KF742543 |  |
|  | KJ680342 | KM624031 |  |  |  |  |  |
| 2008 | EU521709 | EU555439 | EU656143 | EU780073 | EU780074 | EU921254 | 29 |
|  | EU921255 | EU921256 | EU921257 | FJ440338 | GQ174519 | GU325754 |  |
|  | GU450330 | HM038017 | HM035022 | HM038025 | HM038028 | HM038030 |  |
|  | HM038032 | HM038034 | HM776439 | HM776410 | HM779445 | HM776446 |  |
|  | HM776453 | HQ402903 | KC907703 | KF742546 | KJ680345 |  |  |
| 2009 | EU283329 | GU252369 | GU325756 | GU325757 | GU325761 | GU325766 | 37 |
|  | GU325767 | GU325768 | GU325769 | GU325770 | GU370063 | GU370064 |  |
|  | GU450327 | GU450328 | GU450329 | HM003569 | HM003570 | HM102350 |  |
|  | HM776437 | HM776438 | HM776441 | HM776442 | HM776444 | HM776447 |  |
|  | HM776448 | HM776449 | HM776450 | HM776451 | HQ693092 | JF272498 |  |
|  | KC249977 | KF742548 | KF850458 | KF850469 | KJ680349 | KM624035 |  |
|  | KX904949 |  |  |  |  |  |  |
| 2010 | GQ174519 | HQ650833 | HQ693093 | JF272497 | JF272499 | JF682792 | 28 |
|  | JF718784 | JQ806749 | JQ955679 | JX948769 | JX948781 | JX982226 |  |
|  | JX982228 | KC336418 | KC447454 | KC800634 | KC823058 | KF742540 |  |
|  | KF742550 | KF850459 | KF850460 | KF850461 | KF850462 | KJ680343 |  |
|  | KJ680355 | KJ680356 | KM096530 | KX904947 |  |  |  |
| 2011 | JF682793 | JF682794 | JF899334 | JF928002 | JF928003 | JF928004 | 81 |
|  | JF928005 | JF928006 | JN119255 | JN119256 | JN119257 | JN615187 |  |
|  | JN639856 | JN639857 | JQ002672 | JQ413808 | JX274295 | JX406419 |  |
|  | JX406421 | JX406422 | JX406423 | JX406425 | JX406426 | JX678978 |  |
|  | JX682407 | JX912914 | JX912915 | JX945575 | JX945576 | JX945577 |  |
|  | JX948768 | JX948770 | JX948771 | JX948772 | JX948773 | JX948774 |  |
|  | JX948775 | JX948776 | JX948777 | JX948778 | JX948779 | JX948780 |  |
|  | JX948782 | JX948783 | JX948784 | JX948785 | JX948786 | JX982219 |  |
|  | JX982220 | JX982221 | JX982222 | JX982223 | JX982224 | JX982225 |  |
|  | JX982227 | KC800635 | KC800637 | KC800639 | KC800640 | KC800641 |  |
|  | KC800642 | KC800643 | KC800645 | KC800646 | KC823059 | KF742541 |  |
|  | KF850466 | KF850467 | KF850468 | KJ680347 | KJ680348 | KJ680350 |  |
|  | KJ680351 | KJ680352 | KJ680353 | KJ680359 | KJ680361 | KJ680362 |  |
|  | KJ680366 | KJ680370 | KM624033 |  |  |  |  |
| 2012 | JX294717 | JX406420 | JX534236 | JX534237 | KC153106 | KC514987 | 68 |
|  | KC514989 | KC514991 | KC514993 | KC514995 | KC514997 | KC514999 |  |
|  | KC515001 | KC515003 | KC515005 | KC515007 | KC515009 | KC515011 |  |
|  | KC515013 | KC515015 | KC515017 | KC515019 | KC515021 | KC515023 |  |
|  | KC515025 | KC515027 | KC515029 | KC533811 | KC684978 | KC751546 |  |
|  | KC753768 | KC753769 | KC753770 | KC753771 | KC753772 | KC788504 |  |
|  | KC800636 | KC800638 | KC800644 | KC823053 | KC823054 | KC823055 |  |
|  | KC823056 | KC823057 | KC859451 | KC860786 | KF027491 | KF027492 |  |
|  | KF027493 | KF027494 | KF027495 | KF027496 | KF027497 | KF732649 |  |
|  | KF742545 | KF742551 | KJ599673 | KJ680346 | KJ680354 | KJ680357 |  |
|  | KJ680358 | KJ680360 | KJ680363 | KJ680364 | KJ680365 | KJ680367 |  |
|  | KJ680368 | KJ680369 |  |  |  |  |  |
| 2013 | KC527542 | KC821781 | KC821782 | KC821783 | KC821784 | KC821785 | 55 |
|  | KF742544 | KF742547 | KF742549 | KF742552 | KF742553 | KF926650 |  |
|  | KJ139962 | KJ511870 | KJ511871 | KJ511872 | KJ511873 | KJ511874 |  |
|  | KJ511875 | KJ511876 | KJ511877 | KJ596438 | KJ867553 | KJ867554 |  |
|  | KJ867555 | KJ867556 | KM360049 | KM360051 | KM360052 | KM360054 |  |
|  | KM360055 | KM360056 | KM360057 | KM434195 | KM460824 | KM487708 |  |
|  | KM487709 | KM880080 | KM880082 | KM880084 | KP081538 | KP081540 |  |
|  | KP081542 | KP081554 | KP081556 | KP670420 | KP670421 | KU041851 |  |
|  | KU311010 | KU311014 | KU311016 | KU311018 | KU960935 | KX247842 |  |
|  | MG229682 |  |  |  |  |  |  |
| 2014 | KM035760 | KM035761 | KM035762 | KM067384 | KM067385 | KM235959 | 46 |
|  | KM235960 | KM235961 | KM272211 | KM272212 | KM503044 | KM624039 |  |
|  | KM880086 | KM880088 | KP081544 | KP081546 | KP081548 | KP081552 |  |
|  | KP112484 | KP112486 | KP313251 | KP313252 | KP313253 | KP313254 |  |
|  | KP637021 | KP670418 | KP670419 | KU041848 | KU041854 | KU041859 |  |
|  | KU311020 | KU311022 | KU311024 | KU311026 | KU311028 | KU311030 |  |
|  | KU311032 | KU311034 | KU317472 | KU960929 | KX247786 | KX247828 |  |
|  | KX247830 | KX298473 | KY940538 | KY940542 |  |  |  |
| 2015 | KR258797 | KT284886 | KT284887 | KT284889 | KU317474 | KU317476 | 51 |
|  | KU317478 | KU317480 | KU317482 | KU317484 | KU317486 | KU317488 |  |
|  | KU317490 | KU317492 | KU317494 | KU317496 | KU317498 | KU557354 |  |
|  | KU960931 | KU960933 | KU960937 | KU960939 | KX247784 | KX247790 |  |
|  | KX247794 | KX247796 | KX247800 | KX247804 | KX247808 | KX247812 |  |
|  | KX247814 | KX247816 | KX247818 | KX247824 | KX247826 | KX247832 |  |
|  | KX247834 | KX247836 | KX247838 | KX247840 | KX247844 | KX814348 |  |
|  | KX826908 | KX831476 | KX831477 | KX831478 | KX831481 | KX865092 |  |
|  | KY940535 | MK347360 | MK347413 |  |  |  |  |
| 2016 | KX352160 | KX352161 | KX855982 | KX855984 | KX865093 | KX865094 | 44 |
|  | KX867819 | KX867821 | KX981602 | KX981603 | KX981604 | KY305203 |  |
|  | KY347898 | KY425814 | KY659550 | KY659551 | KY659552 | KY659553 |  |
|  | KY659554 | MF589529 | MF589537 | MF677842 | MF679590 | MF679593 |  |
|  | MF679594 | MF679595 | MF679597 | MG182438 | MG182440 | MG732822 |  |
|  | MG732829 | MG893895 | MH046795 | MH055402 | MH055404 | MH055406 |  |
|  | MH055407 | MH055408 | MH055409 | MH055410 | MH055411 | MH055412 |  |
|  | MH465471 | MK347361 |  |  |  |  |  |
| 2017 | MF589523 | MF589524 | MF589528 | MF589538 | MF677844 | MF679591 | 53 |
|  | MF679596 | MG182441 | MG182443 | MG182445 | MG732799 | MG732801 |  |
|  | MG732803 | MG732805 | MG732807 | MG732809 | MG732810 | MG732812 |  |
|  | MG813265 | MH046838 | MH046839 | MH059556 | MH059558 | MH059560 |  |
|  | MH059562 | MH059563 | MH059564 | MH059565 | MH059566 | MH059567 |  |
|  | MH094774 | MH094776 | MH094778 | MH094780 | MH094782 | MH094784 |  |
|  | MH094786 | MH094788 | MH094790 | MH094792 | MH094794 | MH151168 |  |
|  | MH920552 | MH920556 | MH920558 | MH920562 | MH920568 | MH922991 |  |
|  | MH922992 | MH922993 | MH931449 | MK139828 | MK404177 |  |  |
| 2018 | MG786932 | MG786933 | MG786934 | MG798696 | MH593263 | MH620789 | 46 |
|  | MH645914 | MH656967 | MH920560 | MH920564 | MH920570 | MH920571 |  |
|  | MH920575 | MH920576 | MH920580 | MH920581 | MH920582 | MH920587 |  |
|  | MH922989 | MH922990 | MK305877 | MK305879 | MK305883 | MK347358 |  |
|  | MK347362 | MK347373 | MK347383 | MK347388 | MK347391 | MK347393 |  |
|  | MK347398 | MK347401 | MK404181 | MK405698 | MK405700 | MK426837 |  |
|  | MK426839 | MK552324 | MK585076 | MK604480 | MK604482 | MK604487 |  |
|  | MK604489 | MK604494 | MK604496 | MN170531 |  |  |  |
| 2019 | MK987069 | MN196673 | MN258755 | MN258756 | MN258757 | MN258758 | 10 |
|  | MN258759 | MN258760 | MN258761 | MN258762 |  |  |  |

**Table S2. Different PCV2 genotypes in each year. Related to Figure 2.**

| Year | Total No. | genotype | | | | | | | |
| --- | --- | --- | --- | --- | --- | --- | --- | --- | --- |
|  |  | PCV2a | | PCV2b | | PCV2d | | Others | |
|  |  | No. | /% | No. | /% | No. | /% | No. | /% |
| 2001 | 3 | 3 | 100.0 | 0 | 0.0 | 0 | 0.0 | 0 | 0.0 |
| 2002 | 8 | 1 | 12.5 | 5 | 62.5 | 2 | 25.0 | 0 | 0.0 |
| 2003 | 19 | 2 | 10.5 | 12 | 63.2 | 5 | 26.3 | 0 | 0.0 |
| 2004 | 26 | 0 | 0.0 | 21 | 80.8 | 5 | 19.2 | 0 | 0.0 |
| 2005 | 38 | 4 | 10.5 | 30 | 78.9 | 4 | 10.5 | 0 | 0.0 |
| 2006 | 34 | 0 | 0.0 | 31 | 91.2 | 3 | 8.8 | 0 | 0.0 |
| 2007 | 38 | 4 | 10.5 | 25 | 65.8 | 9 | 23.7 | 0 | 0.0 |
| 2008 | 29 | 2 | 6.9 | 20 | 69.0 | 7 | 24.1 | 0 | 0.0 |
| 2009 | 37 | 3 | 8.1 | 15 | 40.5 | 19 | 51.4 | 0 | 0.0 |
| 2010 | 28 | 3 | 10.7 | 9 | 32.1 | 16 | 57.1 | 0 | 0.0 |
| 2011 | 81 | 11 | 13.6 | 15 | 18.5 | 55 | 67.9 | 0 | 0.0 |
| 2012 | 68 | 5 | 7.4 | 14 | 20.6 | 49 | 72.1 | 0 | 0.0 |
| 2013 | 55 | 2 | 3.6 | 17 | 30.9 | 36 | 65.5 | 0 | 0.0 |
| 2014 | 46 | 4 | 8.7 | 9 | 19.6 | 31 | 67.4 | 2 | 4.3 |
| 2015 | 51 | 4 | 7.8 | 15 | 29.4 | 32 | 62.7 | 0 | 0.0 |
| 2016 | 44 | 4 | 9.1 | 13 | 29.5 | 27 | 61.4 | 0 | 0.0 |
| 2017 | 53 | 4 | 7.5 | 14 | 26.4 | 33 | 62.3 | 2 | 3.8 |
| 2018 | 46 | 7 | 15.2 | 14 | 30.4 | 25 | 54.3 | 0 | 0.0 |
| 2019 | 10 | 1 | 10.0 | 2 | 20.0 | 7 | 70.0 | 0 | 0.0 |
